# Nanomaterials in Skin Regeneration and Rejuvenation

**DOI:** 10.3390/ijms22137095

**Published:** 2021-06-30

**Authors:** Emanuela Bellu, Serenella Medici, Donatella Coradduzza, Sara Cruciani, Evzen Amler, Margherita Maioli

**Affiliations:** 1 Department of Biomedical Sciences, University of Sassari, Viale San Pietro 43/B, 07100 Sassari, Italy; ema.bellu@hotmail.it (E.B.); donatella.coradduzza@libero.it (D.C.); sara.cruciani@outlook.com (S.C.); 2 Department of Chemistry and Pharmacy, University of Sassari, Vienna 2, 07100 Sassari, Italy; sere@uniss.it; 3 UCEEB, Czech Technical University, Trinecka 1024, 27343 Bustehrad, Czech Republic; evzen.amler@lfmotol.cuni.cz; 4 Institute of Biophysics, 2nd Faculty of Medicine, Charles University, V Uvalu 84, 150 06 Prague 5, Czech Republic; 5 Center for Developmental Biology and Reprogramming (CEDEBIOR), Department of Biomedical Sciences, University of Sassari, Viale San Pietro 43/B, 07100 Sassari, Italy; 6 Interuniversity Consortium I.N.B.B., Viale delle Medaglie d’Oro, 305, 00136 Roma, Italy

**Keywords:** nanomaterials, stem cells, cellular mechanisms, skin, regenerative medicine

## Abstract

Skin is the external part of the human body; thus, it is exposed to outer stimuli leading to injuries and damage, due to being the tissue mostly affected by wounds and aging that compromise its protective function. The recent extension of the average lifespan raises the interest in products capable of counteracting skin related health conditions. However, the skin barrier is not easy to permeate and could be influenced by different factors. In the last decades an innovative pharmacotherapeutic approach has been possible thanks to the advent of nanomedicine. Nanodevices can represent an appropriate formulation to enhance the passive penetration, modulate drug solubility and increase the thermodynamic activity of drugs. Here, we summarize the recent nanotechnological approaches to maintain and replace skin homeostasis, with particular attention to nanomaterials applications on wound healing, regeneration and rejuvenation of skin tissue. The different nanomaterials as nanofibers, hydrogels, nanosuspensions, and nanoparticles are described and in particular we highlight their main chemical features that are useful in drug delivery and tissue regeneration.

## 1. Introduction

Skin represents the first line of defence of the human body, being exposed to external stimuli, thus playing an essential role against injuries and damages [1,2]. Wound and aging are two processes arising from skin exposure to injuries. For this reason, tissue regeneration and rejuvenation represent fields of interest in clinical practice. Within this context, the use of scaffolds, nanomaterials and bioactive molecules, has been largely employed to support tissue homeostasis.

### 1.1. Aging

Aging in skin arises from two different mechanisms: an intrinsic and an extrinsic aging. These processes are associated with the progressive loss of function and higher stress sensitivity of the involved tissues and cell types, influencing human life and affecting featured phenotypic changes [3]. Intrinsic aging takes place along time, involving tissues of the entire organism. On the other hand, extrinsic aging, also called photoaging, arises from environmental stimuli and is related to lifestyle, nutrition and stress response mechanisms [4]. UV radiation is one of the external stimuli affecting skin, generating photo-oxidative stress on skin cell populations [5]. Then, epidermal layer undergoes keratinocyte turnover to replace damaged elements [6]. However, the dermis is more affected by extrinsic aging, with loss of tone and hyperpigmentation, deeply changing residents cell behaviour [7]. Moreover, the dermal layer mainly comprises extracellular matrix (ECM) whose collagen fibrils and hyaluronic acid organization and production change during aging [8,9]. The reduced amount of ECM fibres results in a diminished elasticity and thickness of the tissue. Within this context, age-specific changes in collagen turnover and production have also been described [10,11].

Stem cells play a crucial role in maintaining tissue homeostasis thanks to their capability to replace damaged cells restoring tissue functions [12]. Stem cells are undifferentiated elements able to replace damaged elements differentiating after specific stimuli [13,14].

For this reason, preserving stem cell plasticity and differentiation capability represents an important goal in tissue regeneration. Nevertheless, skin stem cells (SSCs) are affected by damages as the other cell populations in the tissue, undergoing different changes: they downregulate stemness related genes and lose the capability to replace damaged elements triggering tissue aging [15,16]. Besides stem cells, fibroblasts also accumulate cellular damages leading to a minor ECM production along with aging [17,18,19].

Nanomaterials can stimulate SSCs proliferation and the maintenance of a young phenotype and the modulation of fibroblast gene expression leading to a proper ECM production and guaranteeing a young skin thanks to the photoprotection and the antiaging qualities [20,21].

### 1.2. Wound Healing

The term wound indicates all the damages or disorders of skin due to trauma or therapeutic conditions. Once a wound occurs, skin loses its morphological features and functions in the affected area. These damages can be either acute or chronic, depending on duration. Acute wounds arise from mechanical or physical damages occurring after heat, electricity or chemicals exposition as well as after surgery. This type of wound is rapidly solved when properly managed. On the other hand, chronic wounds are very complex events, with difficult resolution, being often a complication of other diseases like diabetes [22]. The wound area can be colonized by microorganisms leading to making the healing process slower and more complicated [23].

Wound healing comprises a sequence of events involving various cell types and molecules, usually divided into different phases. The first is the inflammatory phase involving platelet and biological signals [24,25].

Then, the proliferative phase occurs, when regeneration restores skin functions. The main actors of these events are stem cells and fibroblasts, that, by migrating to the damaged site, take care of extracellular matrix depot and inflammatory mediators secretion [26,27]. Fibroblasts are responsible for wound closure, from the early inflammatory phase to the final phase comprising extracellular matrix production, essential to restore skin barrier [26]. Additionally, stem cells are involved in skin regeneration, undergoing proliferation and differentiation toward keratinocytes and/or fibroblasts before the final re-epithelization [13,27].

### 1.3. Skin Permeation by Topical Treatments

Products able to counteract age-related complications, ameliorating the wound healing process, are largely used in medicine and cosmesis [28]. However, the intrinsic features of skin provide an efficient barrier against elements coming from the environment, also regulating the flux of chemicals counteracting the transition of hydrophilic and hydrophobics drugs [29].

**Figure 1:**
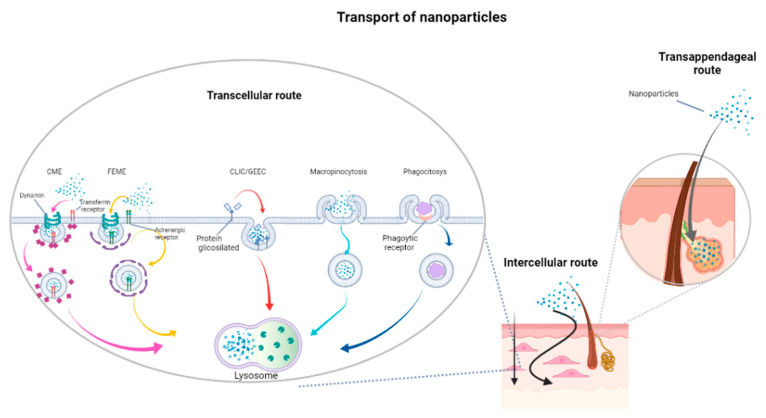
Schematic representation of the three pathways involved in the internalization of molecules through the skin. The image comprises trans-appendageal route, intercellular route and transcellular route. The transcellular route representation is inspired by Rennick et al. [32].

The flux of molecules through the skin occurs by passive diffusion, following a gradient concentration, both via intracellular and extracellular pathways. However, large molecules cannot permeate the skin and the transdermal delivery is still far from satisfying the clinic and cosmetic requests [29]. Indeed, drugs can be carried through the skin by three pathways according to their physical and chemical properties: transappendageal route, trans-epidermal route or transcellular route [30] (Figure 1). Moreover, skin permeability could be affected by several factors, including age [31].

For these reasons, the accomplishment of skin treatments is not only dependent on the molecules, but also on the delivery system and its proprieties. Bioactive molecules, properly delivered, can implement regenerative and rejuvenating processes supported by skin cell populations. Moreover, a suitable formulation can modulate molecule release, increasing drug solubility [20,27,33,34,35,36]. Nanomedicine discloses novel chances for innovative pharmacotherapeutic approaches enhancing the passive penetration and increasing the thermodynamic activity of drugs [37,38].

Currently, cosmetic products as sunscreens, hair products, and skin creams contain nanoformulations [37], and although nanomaterials were first employed in cosmetics thirty years ago with liposomes, the discovery of novel devices is still a leading research trend, recently including different materials and techniques such as wound dressing [39,40].

Nanomaterials (Table 1) are particles with at least one dimension in the range 1–100 nm, showing peculiar properties causing a different kind of interactions with the environment [41] respect to conventional materials. First, they possess a very high surface-to-volume ratio compared to bulky matter, that also determines a change in reactivity respect to their massive counterparts [42]. Second, their size is perfect for an effective interaction with cells, especially when dealing with internalization. Third, domination of some physical phenomena compared to macroscopic materials, like surface tension, embodies in nanomaterials unexpected characteristics unknown or negligible in bulky materials. These characteristics could be, with a great advantage, also utilized in drug delivery systems.

The mechanisms involved in such processes are of a totally different nature with respect to classical molecules. There are two main routes of entry into the cell: direct fusion with the plasma membrane or endocytosis. The main route used by nanoparticles to enter into the cell is endocytosis. To date, there are five main types of endocytosis: (1) clathrin-coated cavity-mediated endocytosis (CME; clathrin and dynamin dependent); (2) endophilin-mediated rapid endocytosis (FEME, a clathrin-independent but dynamin-dependent) ligand-driven rapid endocytosis of specific membrane proteins); (3) clathrin-independent carrier (CLIC)/glycosylphosphatidylinositol-anchored protein, enriched early endocytic compartment endocytosis (GEEC) (clathrin and dynamin independent); (4) macropinocytosis; and (5) phagocytosis (Figure 1) [32].

The excretion processes are also different, being that cells are more prone to retaining nanoparticles than other molecular formulations [43]. Nanomaterials can be functionalised for cell targeting and drug delivery, so that they are able to transport their cargo of useful therapeutics directly to the site where they are needed [44]. For these and many other reasons, nanoparticles represent one of the most promising tools for biomedicine in general, but specifically for skin and wound repair interventions.

## 2. Tissue Regeneration and Rejuvenation Strategies

Nanoparticles formed by a single chemical species, like metals or metal oxides, besides being good antimicrobial agents, are able to exert healing activities on skin lesions and wounds per se. For instance, gold and silver nanoclusters with sizes between 1.1 and 1.6 nm were found to be active in skin repair in rat models in vivo [45]. In vitro tests, on the other hand, evidenced that gold clusters are the most efficient, with better cell uptake and an improved cell proliferation, probably by enhancing cell metabolism. Moreover, they were also able to promote cell migration, a crucial step in skin repair, and an anti-inflammatory activity as ROS scavengers. In general, AuNPs are able to reduce inflammation, promote granulation tissue formation, and skin tissues do not reject them, due to their high biocompatibility [46]. On the other hand, AgNPs can enhance keratocyte and fibroblast proliferation by blocking the respiratory pathways keeping them alive. Moreover, they are able to suppress the innate immune system, a fact that is related to an increased rate of wound healing and decreased rate of the scarring process [47].

As a matter of fact, Acticoat^®^ is the first commercial wound dressing containing silver nanoparticles. The use of silver nanoparticles in skin regeneration finds a significant example in the clinical practice in a case of toxic epidermal necrolysis by carbamazepine administration that caused vesiculobullous lesions and erosions on the 70% of the patient’s body surface. No conventional antibiotic was used to treat skin lesions; only a nanosilver dressing (based on silver nanocrystals) was applied and kept in place using cotton pads and crepe bandages. After five days re-epithelialization was observed and healing was complete after nine days [48]. Another example is nanoceria (spherical cerium oxide nanoparticles, 3–5 nm), that in low doses is able to counteract the effects of UVA-induced photodamage to the skin, favouring cell survival, migration and proliferation [49]. Copper nanoparticles (20, 40 and 80 nm, all spherical in shape) are able to promote endothelial cell migration in a size- and dose-dependent manner while keratinocyte and fibroblast cell proliferation occurs at specific sizes and concentrations. Larger CuNPs (80 nm) increase collagen 1A1 expression in cultured fibroblast cells more efficiently as compared to smaller ones (40 nm). Furthermore, copper nanoparticles can accelerate full-thickness skin wounds healing and increase the formation of new blood vessels in rat models without any drawback [50]. The advantage in using metal or metal-based NPs, especially for their antibiotic properties, lies in the fact that the cationic forms of metals, which are usually the active species, are easily cleared by a series of detoxification mechanisms able to restore the metal homeostasis, thus preventing their therapeutic action. On the other hand, metal NPs are able to circulate in the blood stream or lie inside the cell for a very long time, during which they slowly dissolve, behaving like a small, continuous reservoir of metal ions, for a prolonged action towards their target(s) [51]. Metal nanoparticles are thus suitable for many applications in this research area. Nevertheless, the best results in skin healing and regeneration are obtained with nanocomposite materials, as described in the following sections. Finally, it has to be added that bio- or biocompatible polymers, under proper conditions, can self-assemble to form spherical nanostructures in which drugs can be incapsulated for delivery and controlled release [52].

Summarizing, nanoparticles in wound healing and regeneration are able to exert different actions:

(i) Direct stimulation of cell regrowth;
(ii) Antibacterial activity;
(iii) Drug delivery.

These can be often reunited in a single nanoformulation for a synergistic effect and a faster recovery. All these aspects will be thus discussed and analysed in the next chapters, and are resumed in Table 2.

### 2.1. Nanohydrogels and Nanoparticle–Hydrogel Superstructures

Hydrogels are one of the most promising wound dressing materials because their composition can be tuned to mimic the ECM and provide a moist environment for tissue regrowth, which in turn promotes re-epithelialisation through epithelial cell migration. In fact, they are composed of a 3D crosslinked polymeric network able to withhold large amounts of water while retaining its structure after swelling [67]. This provides an elasticity close to that of the surrounding tissues, and a permeability to oxygen that prevents the growth of anaerobic bacteria. Moreover, hydrogels often promote haemostasis, cell migration and proliferation, thus accelerating the process of healing. Their formulation can be studied to allow injectability, so that they can fill irregular wounds and adhere more tightly to the tissue walls.

There are different ways in which a hydrogel-based nanomaterial can be prepared. Among them, the proper aggregation of polymers (polysaccharides, hyaluronic acid, chitosan, etc.) can lead to the generation of nanohydrogels in the form of nanofilms, nanoparticles or nanofibers [52]. Nanofibers offer a better choice for skin tissue engineering scaffolds because they are able to imitate the properties of biological tissues (vide infra) [68]. Nanoparticle–hydrogel superstructures, on the other hand, are new materials prepared by embedding nanoparticles in hydrogels to produce nanoplatforms with highly tunable properties and a wide range of applications [69]. In fact, when small quantities of nanosized materials are added to a polymer matrix, the performance of the resultant material is improved to an extraordinary level.

Typical components of hydrogels for wound dressings are polyvinyl alcohol (PVA), sodium alginate (SA), cyclodextrin (CD), hyaluronic acid (HA), polyacrylic acid (PAAc), polyacrylamide (PAAm), polycaprolactone (PCL), polyethylene glycol (PEG), polylactic acid (PLA), polyvinyl pyrrolidone (PVP), polyvinyl acetate (PVAc), collagen, pectin, chitin, and many others [70]. Biodegradable materials are obviously preferred since they degrade along with new tissue formation. However, their use as such in skin tissue regeneration is rather limited due to poor mechanical properties. This can be circumvented by incorporation of nano- and biomaterials able to reinforce their structure and convert hydrogels into multifunctional nanocomposites with a series of other advantages [68]. In fact, their formulation can be properly modified to exert different functions at the same time, as it often happens also with other nanomaterials for skin regeneration.

#### 2.1.1. Antibacterial Action

This was traditionally achieved by the addition of conventional antibiotics (such as sulfadiazine, whose properties have been addressed in a dedicated section, vide infra), but with the advent of nanomaterials this role has been taken by metal or metal-based nanoparticles (such as gold [71], silver [59,60,61], copper, zinc oxide, copper oxide or sulphide [54,62,63]. Metal nanoparticles (e.g., AuNPs and AgNPs) exert their antimicrobial activity by a series of mechanisms involving direct interaction with the negatively charged bacterial cell membrane, its disruption, and subsequent leakage of the inner cellular material [51]. Once the NPs get inside the cell, they can further damage it by impairing DNA or other cellular components. Noble metal NPs can also generate ROS to cause additional harm to the bacteria [54], and while AuNPs are also able to inhibit the ATP synthase, further lowering the ATP levels and leading to cell death from a downfall in energy metabolism, silver in particular can set up a sequence of events that cannot be simultaneously counteracted by adaptive measures. For such reasons, AgNPs are not able to induce resistance in any bacterial strain tested to date, except P. stutzeri that was isolated in silver mines. In a similar way, CuS NPs, which are well established non-toxic nanomaterials, carry out their antibiotic action by damaging the cellular membrane and producing ROS [64]. Zinc ferrite (ZnFe_2_O_4_) NPs antimicrobial activity is exerted via multiple mechanisms that include all those previously cited: cell membrane damage, protein leakage and reactive oxygen species generation [65].

On the other hand, metal oxide NPs are more prone to exert their antibiotic activity through photocatalysis and can be activated by proper wavelength irradiation. In fact, the ultraviolet portion of light is able to produce free radicals when impacting with the metal oxide nanoparticle surface, such as oxygen and hydroxyl radicals, that can rapidly kill bacteria [72,73]. Thus, the presence of metal or metal-based NPs in the formulation of hydrogels, as well as in other composite material used in wound dressing, is highly desirable [54].

Another common component in hydrogels possessing intrinsic antimicrobial activity is a biocompatible, biodegradable and non-toxic natural polymer, chitosan, due to the interaction of its positive charges with the negative charges on the cellular membranes of bacteria. Chitosan is often employed in the preparation of biomaterials for wound repair, although its poor mechanical properties and low water solubility limit its applications. Therefore, it has to be properly modified or functionalized, for example by introduction of a quaternary ammonium moiety onto its backbone [74,75].

Antimicrobial peptides (AMPs) are also employed in the formulation of hydrogels [75,76,77]. They are cationic and amphiphilic (hydrophilic and hydrophobic) α-helical peptide molecules that represent one of the first-line defences of the host against bacteria, being essential components of the innate immune system in various species, including humans, animals and plants. Cationic peptides interact with the negatively charged bacterial cell membrane, changing its potential and leading to its disruption, with a series of related event that can result in cell death. Natural AMPs are rather effective, but possess a series of drawbacks that limit their use in therapy: they have a short half-life, can be toxic and can cause haemolysis, for instance [78]. Thus, adequate modification of natural AMPs or synthesis of new peptides with the right qualities are required to exploit their antibiotic action in nanohydrogels and other nanomaterials.

#### 2.1.2. Antioxidant Properties

Hydrogels can be formulated to host an antioxidant species to decrease damages caused by ROS, thus ameliorating the process of wound healing. Such molecules are usually polyphenols and their derivatives, but also other compounds, from both natural and synthetic sources, have been employed with remarkable results. Ceria [79], curcumin [80], hydroxycinnamic acid derivatives, such as p-coumaric and ferulic acids [81], tannic acid [82], propolis [83], natural plant extracts [84,85,86], anthocyanins [87] and many other phytochemicals within nanocomposites have been tested for their ROS scavenging activities with relevant effects, especially in the case of diabetic chronic wounds, where over-production of ROS can impair angiogenesis and results in continuous inflammation [88,89,90].

#### 2.1.3. Drug Release

Hydrogel nanocomposites may function as reservoirs for a controlled release of drugs and other molecules necessary for an optimal wound healing. For instance, scar suppression can be achieved by slow pulsatile release of an inhibitor of the transforming growth factor-β (TGFβ) [91]. APOSEC is a novel, innovative drug, able to promote healing of diabetic foot ulcers, and it was included in a hydrogel tested in the phase I/II study MARSYAS II with successful results [92]. Pro-angiogenic drug deferoxamine (DFO) was inserted in a dual-crosslinked mussel-inspired hydrogel with antibacterial and angiogenic properties applied in the treatment of chronic infected diabetic wounds, and was delivered via pH modification [93]. The efficacy of drug release in nanoformulations is equally well documented [68,94].

### 2.2. Nanofibers and Scaffolds

Nanofibers are fibres featured by high surface area, low basis weight, with a minimum aspect ratio of 1000:1, high strength rate and high content of small pore size [95,96,97,98,99]. These nanomaterials are particularly interesting for their features resembling the extracellular matrix and stimulating tissue regeneration. Nevertheless, nanofibers are known as an excellent antimicrobial device, protecting and covering skin, being prepared for medical application conforming all safety rules [100]. Nowadays, nanofibers are used for skin regeneration and rejuvenation as scaffolds, and for drug delivery. They can be composed of natural, synthetic and mixed polymers, each showing peculiar characteristics [101]. Natural polymers are highly biocompatible, non-toxic, biodegradable, often possess antibiotic activity per se, and can elicit skin contraction during the process of wound healing, ameliorating wound treatment, [102,103]. On the other hand, synthetic polymers can be modulated and modified during their preparation to meet specific requirements for each case of application. This leads to a control in their composition, molecular weight, crystalline structure, chemical properties and mechanical features that allow high reproducibility for these materials. Hybrid polymers represent a successful union of these aspects, resulting in the best performances recorded for nanofibers and the relative scaffolds with a faster wound healing respect to other nanomaterials [104].

Nanoporosity, together with other prominent features in nanofibers, can be controlled during the process of preparation [105,106]. The most common way is through a technique called electrospinning [107]. In such way, scaffolds are produced that can be formed by different non-woven nanofibers, disposed in random orientations, and with different shape.

Currently, nanofibers of electrospun poly-ε-caprolactone (PCL) and polyvinyl alcohol (PVA) have been described as safe, well tolerated and effective. They can be used as a smart skin cover dressing during wound healing process alone or in combination with bioactive molecules [20,108]. Among the different subjects functionalized in nanofibers, platelets have been recently described, to create a device delivering platelet-derived bioactive molecules able to improve melanocyte proliferation. This result is interesting in the management of skin vitiligo process, involving melanocytes [109]. PCL scaffolds with adhered platelets are also able to stimulate skin cell proliferation, promoting cell propagation, and metabolic activity in all skin-associated cell types [110]. Within this context nanofibers are emerging as a good candidate for diabetic foot ulcer treatments [111]. Moreover, nanofibers produced with PCL and gelatine displayed a faster healing rate, when exopolysaccharide was loaded into a nanofiber on full-thickness wounds in rat models [112]. Furthermore, PCL nanofibers loaded with silver and magnesium ions showed antibacterial activity and pro-angiogenesis function for wound repair on vascular endothelial cells in vitro [113]. When Ag-doped magnetite nanoparticles were used, the resulting PCL scaffold had enhanced cell adhesion and growth [114]. By increasing the silver concentration in the magnetite phase a parallel increase in the viability of human melanocytes and antibacterial activity against E. coli and S. aureus was obtained, together with an improvement in the skin wound healing rate in rats. No abnormalities in the dermal and epidermal tissues were evidenced after 10 days in the treatment group. A PCL-based three-layer nanofiber containing also chitosan and polyvinylalcohol was loaded with melatonin (20%) to afford a new nanomaterial with high water uptake (around 400% after 24 h) and cell adhesion, resulting in a fast wound healing, with complete regeneration of the epithelial layer, decrease in inflammatory cells, collagen synthesis and remodelling of wounds [115]. A combination between poly-ε-caprolactone nanofibers with embedded magnetic nanoparticles is able to accelerate the proliferation of mesenchymal stem cells (MSCs) in vitro, [116]. Moreover, PCL nanofibers combined with natural extracts are able to protect mesenchymal stem cells of the skin (SSCs) from UV induced aging, suggesting a role in skin rejuvenation [20].

**Figure 2:**
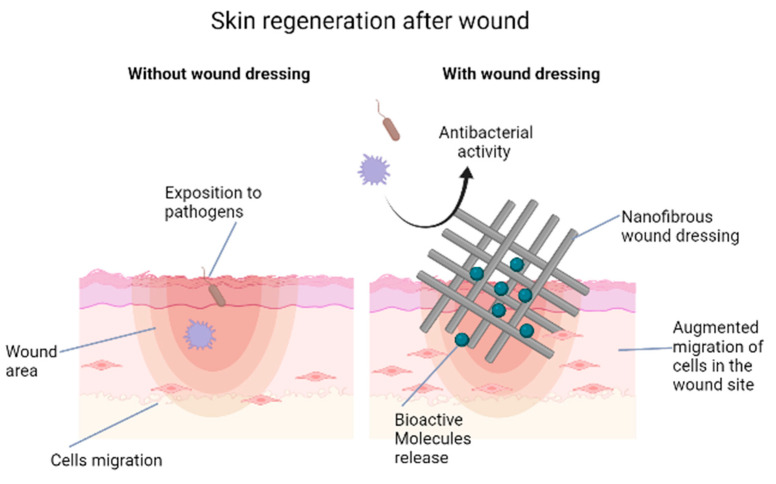
Skin regeneration after wound process with or without wound dressing. The nanofibers mimic the ECM and provides a proper environment for tissue growth, promoting re-epithelialisation and cell migration. They can be used in combination with bioactive molecules (functionalization) improving cell adhesion and proliferation, showing also antimicrobial activity.

Electrospun wound dressing can be prepared to be loaded with different therapeutic or antimicrobial agents to improve wound healing. Unfortunately, the currently available wound dressings are not able to fulfil all the expectations because they are unable to restore the structural and functional properties of the native skin [117]. Nevertheless, nanofibers are presently being studied and improved for their application in tissue regeneration, both in wound healing and beauty treatment. In the former case, nanofibers are exploited to deliver drugs or other biological components (e.g., growth factors [118] and stem cells [119] to the wound site; in the latter, facial masks made of nanofibers are actually used for the release of collagen and vitamins on human skin [41]. A schematic example of a nanofiber-based wound dressing could be found in Figure 2. Among the many examples of drug-loaded nanofibers, a cerium-doped bioactive glass in a chitosan/polyethylene oxide nanofiber showed remarkably high antibacterial action against both Gram-negative and Gram-positive bacterial strains [119]. Heparin has high affinity for many growth factors that are crucial biological mediators in the wound healing mechanism. Poly(lactic-co-glycolic acid)-encapsulated heparin nanoparticles incorporated into sericin/gelatine (1:2) nanofibers are able to combine the controlled release of the drug to the skin regeneration properties of the two biopolymers, resulting in good nanofiber morphology, high water retention and low degradation of the scaffold that made it an interesting nanomaterial for heparin topical delivery [120].

### 2.3. Antiscar Action

Wound healing is associated to scar formation that sometimes can be abnormal (hypertrophic or keloidal scars) [121]. Hydrogel nanocomposites and electrospun nanofibers, both natural or synthetic (PCL, PLGA, PVA, collagen, chitosan, silk fibroin, alginate/PVA, etc., also associated to noble metals nanoparticles or other therapeutics) can help reduce the scarring process. For instance, PLGA nanofibers transformed to contain carboxylic acid groups exhibited enhanced fibroblast cell adhesion and proliferation, desired properties for a correct wound healing [122]. In fact, this functionalization can enhance the binding of nanofibers to collagen or gelatine, which are key proteins found in the extracellular matrix, and this can improve cell adhesion and proliferation [123]. Moreover, electrospun systems such as alginate/PVA nanofibers are able to induce scar-free wound healing since they can retain humidity to maintain a moist wound microenvironment, as it has been demonstrated that when a wound is kept moist the scar tissue formation is decreased [124]. Silk fibroin electrospun fibres possess the same properties as fibroin, which is known to have anti-inflammatory action and a remarkable anti-scarring potential. Additionally, in fact, such nanofibers showed decreased levels of pro-inflammatory IL-1α in skin tissues, together triggering collagen formation, which arranged itself within the wound in a way similar to normal skin instead of a scarring composition [125]. Other factors that can reduce scar tissue formation are a more rapid and efficient cell migration, or a block of fibroblast differentiation into myofibroblasts, that can be induced by proper formulation of the nanofibers used to treat the wound [126]. It is evident that nanofibers or nanofiber-based composites can have an important role in the proper growth of scar-free skin tissues.

## 3. Silver Sulfadiazine Nanomaterials

Silver sulfadiazine (SSD) is a drug based on a silver complex with an antibiotic of the sulphonamide family. Its introduction in clinics dates back to the early 1960s to treat burn wounds, but it was also marginally used to heal skin wounds in general. Its current formulation in the therapy of second- and third-degree burns is a 1% cream for topical administration [53]. The clinical application of silver sulfadiazine has been controversial since two Cochrane systematic studies (2010 and 2013) questioned its efficacy in burn wounds treatment and concluded that the evidence collected was not sufficient to establish it. Moreover, SSD exhibits delayed wound healing due to fibroblast toxicity. Nevertheless, silver sulfadiazine is still considered by many clinicians as a crucial topical drug in the management of burn patients [53]. To improve its efficacy and optimize its administration, nanotechnology has been exploited. Several nanoformulations have been prepared to obtain better results in the treatment of wounds, from either burns or other causes.

Silver sulfadiazine nanosuspensions were obtained with 0.5% SSD in 6% Cremophor EL and 4% Lauroglycol 90, while nanogels were made with 0.5% SSD in 1% Carbopol 974 P [127]. Nanosuspensions were faster than nanogels in releasing SSD, and although they both had the same activity against several bacterial strains as an SSD solution in vitro, the in vivo application of such 0.5% SSD nanoformulations had a higher efficacy in wound healing compared to commercially available 1% topical creams.

A different composition for the administration of SSD could be found in the preparation of a non-propellant-based foam containing sulfadiazine and pectin capped “green” AgNPs. The foam had an average globule size of <75 nm, caused no skin inflammation and showed a good recovery of the burnt tissues, with an evident regeneration of the derma in superficial second-degree (partial thickness) burn wounds [128].

Polyvinyl alcohol/carboxymethylcellulose/silver sulfadiazine composite nanofibers (PVA/CMC/SSD) were prepared and found to be active in the treatment of excision wounds, leading to rapid healing in rabbits. The nanocomposite also displayed good antibacterial activity against P. aeruginosa and S. aureus, being at the same time nontoxic against fibroblasts [129].

An important aspect in skin lesions that has not been mentioned so far, but can be fundamental for a proper healing, is the presence of biofilm. In fact, bacteria can grow in two ways: the planktonic and the sessile forms. In the former, single bacterial cells are free to move, while in the latter they aggregate in colonies that can adhere to both living and non-living surfaces [53]. This may happen also in chronic wounds, and the occurrence of biofilms on damaged skin is always a concern, since such colonies are surrounded by a matrix of extracellular polymeric substances (mainly polysaccharides, but also small amounts of proteins, enzymes, DNA, and RNA) that shields the bacterial cells from the outer environment and prevents antibiotics from reaching them. For this reason, drug treatment of biofilms requires high doses of antibacterial agents for prolonged times. Silver sulfadiazine was found to be particularly active also against biofilms, and SSD nanoformulations exhibits the same properties. For this reason, a chitosan gel containing solid lipid nanoparticles of silver sulfadiazine (SSD-SLNs) and deoxyribonuclease-I (DNase-I) was designed with the double aim of winning biofilm resistance and decreasing SSD fibroblast cytotoxicity [66]. DNase-I seems to help the antibiotic agents overcome biofilm infections via hydrolysis of the extracellular DNA, responsible for biofilm adhesion, and the optimal action of the SSD-SLNs/DNase-I nanoformulation was evidenced by the inhibition of nearly 97% of biofilm of Pseudomonas aeruginosa in comparison to SSD with DNase-I only (around 83%). Moreover, the new nanomaterial was less cytotoxic to fibroblasts than SSD alone and showed faster wound healing compared to other SSD and SSD-SLNs preparations. Of course, other nanomaterials showed to be effective against biofilms in wounds, but they are all manly based on silver nanoparticles, either alone or in combination [130,131,132,133].

## 4. Nanoformulations for Skin Care and Anti-Aging Products

Nanotechnology has found wide application in the field of skin care formulations for at least forty years, being this probably one of the first areas of diffusion of nanomaterials in customer products, that started with the introduction of liposomes in moisturizing creams. TiO_2_ and ZnO NPs have been employed for decades as UV inorganic filters in sunscreens, with a safe and effective protection against noxious sun rays and in the prevention of skin cancer, so effective that their ability to block solar radiation was correlated to a decrease of vitamin D production, with contrasting results (read for instance [134,135,136]). Nowadays, the use of nanomaterials in antiaging products is mainly directed towards the delivery and controlled release of pharmaceuticals and cosmeceuticals for different purposes, such as anti-radical action, stimulation of collagen production, protection of skin components, etc. In this perspective, a series of nanocarriers have been employed, especially those based on lipidic materials, starting from liposomes, of course, to include solid lipid nanoparticles (SLN) and nanostructured lipid carriers (NLC), or nanoemulsions. Other useful NPs in cosmetics are dendrimers, nanocrystals, carbon nanotubes, niosomes, nanopolymers, etc. The properties and uses of these nanomaterials have been recently reported in detailed and extensive reviews [137,138] and thus will not be further discussed. As carriers, the nanodevices were loaded with active pharmaceutical ingredients, vitamins or pro-vitamins, polypeptides, plant extracts, essential oils, antioxidant molecules (including enzymes and coenzymes), drugs, etc. In this way, the targeted delivery of active molecules and their selective and controlled release, together with increased permeation of skin by NPs, improved the performances of cosmetic products and the results obtained were superior to classic formulations. The nanocarriers, in fact, can pass through the stratum corneum and exert their action either translocating inside the skin without degradation, or alternatively they can undergo degradation close to the skin surface where the encapsulated therapeutic compounds can be released and then penetrate into the skin layers [138].

The cosmetic industry took advantage of the progresses in nanotechnology to expand its boundaries in personal care and antiaging areas of sales. Several companies have already commercialised products containing dendrimers (such as artificial skin tanning agents, mascaras, nail polish, etc.) or metal nanoparticles [137]. In general, metal-based NPs (Au, Ag, CuO, ZnO, etc), as previously discussed, have been employed in nanocosmetics due to their assessed antibacterial activity and promotion of wound healing (vide supra). Gold nanoparticles, especially, have been introduced in moisturizers, sunscreens, eye creams and lip balms for other properties. Many spas and beauticians offer facial masks and treatments based on AuNPs for skin rejuvenation [55]. It has been demonstrated that gold nanoparticles are able to reduce wrinkles [56], improve skin brightening, promote skin healing, have a cleansing effect, reduce inflammation and ROS damage, provide anti-bacterial action, slow down collagen depletion [57] and elastin degradation [58]. Nevertheless, concerns have been expressed about a safe use of gold-based nano-cosmeceuticals, since it has been demonstrated that Au nanosheets in cosmetic creams are able to quantitatively permeate into the skin epidermis, dermis and subcutaneous layer after a ten-day cutaneous exposure [139]. The same study also showed that Au nanosheets are not able to enter the systemic circulation, though, but can decrease the cell viability of keratinocytes and induce a low level of apoptosis or necrosis of keratinocytes and skin dermal fibroblasts. These findings, again, raise the issue of nanoparticle safety. It seems, though, that biosynthesised AuNPs may have higher biocompatibility and less side effects [53]. In any case, the subject of NPs safety still remains open.

## 5. Considerations on the Toxicity of Nanomaterials in Wound Dressings

The outstanding properties of nanoparticles in the biomedical field, especially those connected to skin treatments, are undeniably crucial in the development of new, advanced materials for these applications. Nevertheless, concern has been expressed during the last decades over NPs toxicity, but with no clear results or conclusions [140]. Due to their dimensions, nanoparticles are able to interact with matter in a way that is completely different respect to the corresponding bulk materials, and they can exert unexpected effects on the living organisms, including humans. NPs size allows their effective interactions with cells, either eukaryotic or prokaryotic. Metal NPs show remarkable antibacterial and anticancer activity, depending not only on their dimensions, but also on their shape, coating/capping/functionalization, solubility, etc. Several studies demonstrated they can also interact negatively with healthy cells, thus causing toxicity and damage based on the same mechanisms through which they exert cytotoxicity [141]. Many studies, on the other hand, found NPs are effective against cancer and bacterial cells, but safe when tested on normal ones. The fact that, after two decades since their introduction in biomedical research, there is still no clear evidence on one case or the other is probably also due to the impossibility of a sensible comparison among different results. In fact, there are too many factors determining NPs toxicity, so that there is no real and effective standard to allow a significant comparison for sound conclusions. What emerged, though, as a general trend, is that particles too small, around 10 nm, are very active against cancer and bacteria, but scarcely biocompatible, being able to induce haemolysis, for instance. Nevertheless, other researcher found that very tiny NPs (<2 nm) can be cleared more easily respect to bigger ones (generally through kidneys) [142], resulting in a decreased toxicity [143]. Shape is important too, as spheroidal nanoparticles can circulate in body fluids for a shorter time respect to non-spherical NPs, thus reducing their interaction with healthy cells. Finally, the outer layer, where a coating of organic molecules (stabilizers, capping agents, bioreductants, etc.) can be found, has been indicated as one of the factors responsible for their intrinsic toxicity [144,145], with citrate-covered NPs more toxic than biogenic or PEGylated NPs.

Skin has been generally considered as a strong barrier to the penetration of nanoparticles in the body, although different papers recently appeared in the literature showing that this is not always true [139]. Damaged skin, such as in burns and wounds, is more penetrable by NPs as compared to healthy derma, therefore the formulation of wound dressings and nano-cosmetics should be carefully designed and thoroughly tested in the respect of nanotoxicity [146]. Moreover, even a healthy skin can suffer from NPs aggressive action [147] and this should always be considered when dealing with new topical treatments.

## 6. Conclusions

Wound healing and skin rejuvenation still represent a difficult challenge in regenerative medicine; nevertheless, the application of nanotechnology has given a huge contribution to the progress in this field, and the nanomaterials discovered during the last years paved the way for novel approaches in wound treatment, tissue regeneration, or to counteract aging related morphogenetic changes.

The nanodevice-mediated controlled drug delivery, protection of the affected areas, biocompatibility, antibacterial activities, etc. have allowed a great expansion in the use of these innovative products alone or in combination, conjugating high performances, ease of administration and safety. The use of nanotechnologies thus represents a great potential and could get concrete advantages in improving wound healing and rejuvenation supporting skin cell populations during adverse conditions and implementing the therapeutic options already used in clinic.

## Figures and Tables

**Table 1:** Illustration of the structure and the materials mainly used in the field of skin regeneration and rejuvenation.

Representative Image of Nanomaterial	Name	Materials Used in Skin Regeneration
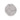	Nanocrystal	Silver, gold, carbon, polymers, etc.
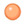	Nanoparticle	Silver, gold, copper, zinc oxide, copper oxide, sulfide, etc.
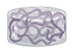	Hydrogel	Polysaccharides, hyaluronic acid, chitosan, polyvinyl alcohol, sodium alginate, cyclodextrin, polyacrylic acid, polyvinyl pyrrolidone, polyvinyl acetate, collagen, pectin, chitin, etc.
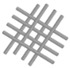	Nanofiber	Polycaprolactone, polyethylene glycol, polylactic acid, polyvinyl pyrrolidone, etc.

**Table 2:** Nanoparticles mainly used for skin regeneration and rejuvenation, and their functions.

Nanoparticle	Description	Function/Use
Gold and silver nanoclusters	Size between 1.1 and 1.6 nm	Skin repair in rat models in vivo [45]. Enhance cell proliferation in vitro and full thickness wound healing [50].
AuNPs	Biosynthesised AuNPs are highly biocompatible and have less side effects [53]	Reduction of inflammation, promotion of granulation tissue formation [46]. Antimicrobial activity [54]. Skin rejuvenation properties [55] including ability to reduce wrinkles [56] improve skin brightening, promote skin healing, have a cleansing effect, reduce inflammation and ROS damage, slow down collagen depletion [57] and elastin degradation [58].
AgNPs		Enhance keratocyte and fibroblast proliferation, suppress the innate immune system increasing wound healing rate and decrease the scarring process rate [47]. Antimicrobial activity [59,60,61].
Nanoceria	Spherical cerium oxide nanoparticles, 3–5 nm	In low doses are able to counteract the effects of UVA-induced photodamage, favouring cell viability, migration, and proliferation [49].
Copper nanoparticles (CuNPs and CuS)	20, 40 and 80 nm, all spherical in shape	Promotion of size- and dose-dependent endothelial cell migration and proliferation, accelerate full-thickness skin wounds healing. Increased collagen 1A1 expression in vitro and increased formation of new blood vessels in rat models [51]. Antimicrobial activity [54,62,63,64].
Zinc ferrite (ZnFe_2_O_4_)		Antimicrobial activity via multiple mechanisms [65].
Silver sulfadiazine		Antimicrobial activity in particular against biofilms [66].
